# Evaluation of Intraoperative and Postoperative Blood Cell Salvage Use in Cardiac Surgery with Cardiopulmonary Bypass

**DOI:** 10.21470/1678-9741-2024-0244

**Published:** 2025-05-23

**Authors:** Marco Antonio Araújo de Mello, Laís da Silva Pereira-Rufino, Antonio Alceu dos Santos, Nelson Americo Hossne Junior, Carlos Eduardo Panfilio, Albert Schiaveto de Souza, Isabel Cristina Céspedes

**Affiliations:** 1 Instituto de Biociências, Universidade Federal do Mato Grosso do Sul - UFMS, Campo Grande, Mato Grosso do Sul, Brazil; 2 Department of Morphology and Genetics, Disciplina de Genética, Escola Paulista de Medicina, Universidade Federal de São Paulo - UNIFESP, São Paulo, São Paulo, Brazil; 3 Department of Clinical and Experimental Oncology, Escola Paulista de Medicina, Universidade Federal de São Paulo - UNIFESP, São Paulo, São Paulo, Brazil; 4 Department of Surgery, Disciplina de Cirurgia Cardiovascular, Escola Paulista de Medicina, Universidade Federal de São Paulo - UNIFESP, São Paulo, São Paulo, Brazil; 5 Escola da Saúde, Universidade Municipal de São Caetano do Sul - USCS, São Caetano do Sul, São Paulo, Brazil

**Keywords:** Cardiopulmonary Bypass, Erythrocytes, Infections, Hemoglobins, Length of Stay, Hematocrit

## Abstract

**Introduction:**

Blood transfusion is associated with adverse clinical and surgical outcomes. Strategies like the Patient Blood Management program, which includes blood cell salvage, contribute to reducing the use of blood components. Blood cell salvage is very useful in heart surgeries where the patient's blood loss can be massive.

**Objective:**

The present study aimed to evaluate the impact of using the blood cell salvage in the intraoperative and postoperative periods (up to 24 hours) on the hemoglobin and hematocrit values, transfusion of red blood cells, infection rates, and postoperative length of stay in patients undergoing cardiac surgery with cardiopulmonary bypass.

**Methods:**

Forty-one patients who underwent cardiac surgery with cardiopulmonary bypass according to the inclusion criteria were selected in an observational study and separated into two groups: with the use of the blood cell salvage group (BCS, n = 21) and without the use of the blood cell salvage (WBCS, n = 20).

**Results:**

Patients in the group using blood cell salvage had higher postoperative hemoglobin (P = 0.018) and postoperative hematocrit levels (P = 0.009), lower consumption of red blood cells in the postoperative period and hospital discharge (P < 0.001), shorter postoperative length of stay (P = 0.020), and lower infection rates (P = 0.009).

**Conclusion:**

Patient Blood Management strategies, particularly the use of blood cell salvage in the intraoperative and immediate postoperative periods of patients undergoing cardiac surgery with cardiopulmonary bypass, are associated with less use of blood components and consequently better clinical outcomes.

## INTRODUCTION

**Table:** 

Abbreviations, Acronyms & Symbols	
BCS	= Blood cell salvage
CI	= Confidence interval
CPB	= Cardiopulmonary bypass
EuroSCORE	= European System for Cardiac Operative Risk Evaluation
Hb	= Hemoglobin
HD	= Hospital discharge
Ht	= Hematocrit
IO	= Intraoperative
IPO	= Immediate postoperative
PBM	= Patient Blood Management
PO	= Postoperative
RBC	= Red blood cells
SE	= Standard error
WBCS	= Without the use of blood cell salvage

Excessive bleeding is a relatively common complication in cardiac surgery with cardiopulmonary bypass (CPB), increasing morbidity and mortality. Prolonged blood contact with the artificial surface of the CPB, high doses of heparin/protamine, hypothermia, and extensive surgical trauma contribute to coagulation disorders and, consequently, bleeding during the intraoperative (IO) and postoperative (PO) periods^[1]^. In this context, approximately 20% of patients have significant bleeding, and 5% require surgical reintervention for hemostatic revision, thus increasing surgical time, length of stay, and treatment expenses^[2]^. Cardiac surgeries generally account for high rates of blood transfusion, particularly red blood cells (RBC), varying between 40% and 90%^[3^,^4]^. Blood transfusion has been independently associated with direct and indirect risks, including immunomodulatory/inflammatory effects and circulatory reactions, leading to an increase in infection rates, prolonged length of stay, and high morbimortality^[5^-^7]^. In this setting, therapeutic strategies like Patient Blood Management (PBM) have gained widespread use. PBM is a multidisciplinary program involving clinical and surgical strategies as therapeutic options for blood transfusions, focusing on the patient’s own condition. This encompasses the treatment of preoperative anemia or coagulopathies (first pillar); bleeding/blood loss minimization during the IO/PO periods (second pillar); and changes in the medical concept of anemia beyond hemoglobin (Hb) or hematocrit (Ht) parameters and strengthening the patient's own physiological reserves, especially in the PO period (third pillar)^[8^-^10]^. As a result of that, the use of blood cell salvage (BCS) (second pillar) for cardiac surgeries has been recommended^[11^,^12]^. BCS in the IO period in cardiac surgeries is safe and effective in reducing blood loss and transfusion^[12]^. Furthermore, BCS in the IO period should always be considered when significant blood loss (500 mL) is predicted. Moreover, the blood collected from the chest drain in the immediate PO (IPO) period can also be returned to the patient after washing and filtration with the BCS device. However, only a few studies have demonstrated the use of BCS in PO thoracic chest drains^[13^,^14]^. In addition, despite the reduction in RBC transfusion with the use of BCS, there is few data about the association between its use and improvement in patients’ clinical outcomes^[11^,^15]^. Thus, the present study aimed to evaluate the impact of using the BCS in the IO and IPO periods (up to 24 hours) on the Hb and Ht values, transfusion of RBC, infection rates, and PO length of stay in patients undergoing cardiac surgery with CPB.

## METHODS

### Participants

All individuals signed the informed consent form. The study was approved by the Research Ethics Committee of the Universidade Federal do Mato Grosso do Sul - UFMS (Campo Grande, Mato Grosso do Sul, Brazil) (nº 6.081.432) and conducted within government ethical standards for clinical research and the principles of the Declaration of Helsinki.

Forty-one patients referred to cardiac surgery and admitted to three different hospitals (Hospital Universitário Maria Aparecida Pedrossian - HUMAP, Brazilian public network [Sistema Único de Saúde - SUS], and Hospital UNIMED and Hospital Caixa de Assistência dos Servidores do Estado de Mato Grosso do Sul - CASSEMS, private network) in Campo Grande (Mato Grosso do Sul, Brazil) were invited to participate in the study. The patients were selected for an observational study and separated into two groups: with the use of the BCS (BCS group) and without the use of the BCS (WBCS group). All patients were operated on by the same surgeon, ensuring the same conditions for all patients.

Patients of both sexes and aged over 18 years were included without distinction of surgical cause. The exclusion criteria were severe liver disease, nephropathy requiring dialysis, decompensated diabetes mellitus (glycated Hb > 18%), limiting peripheral arterial disease or severe psychiatric disorder, and patients who received blood transfusion in the last 30 days. Patients who showed evidence of local or systemic infection in the first 48 hours after surgery and/or progressed to acute conditions (vasoplegic, cardiogenic, or septic shock) were excluded from the study. The European System for Cardiac Operative Risk Evaluation (EuroSCORE) II was used to assess the risk of death. All patients were followed for one year postoperatively.

### Surgical Procedure

Patients in both groups underwent cardiac surgery with CPB with partial hemodilution and hemoconcentration (20% - 30%), in addition to standardization of the IO cannulation technique, systemic heparinization, cardioplegia, and aortic clamping during the IO period. The American Society of Anesthesiologists criteria for anesthetic induction, maintenance, and recovery were followed, with standardization of pharmacological management.

The access route used was median sternotomy using systemic heparinization (300 U/kg) to obtain an activated clotting time > 400 seconds (controls were performed every 60 minutes). CPB circuit was filled with 1500 mL of plasma Lyte (pH 7.4; Baxter; Deerfield, Illinois, United States of America), using an adult membrane oxygenator (LivaNova Inspire™ 8F M - LivaNova; London, United Kingdom). After aorta cross-clamping, cardioplegic solution administration for myocardial protection was the Del Nido solution (Plasma-Lyte A [1000 mL]; sodium bicarbonate 1 mEq/mL [13 mL]; mannitol 20% [16.3 mL]; magnesium sulfate 50% [4 mL]; lidocaine 1% [13 mL]; potassium chloride 2 mEq/mL [13 mL]) with 1000 mL infusion into the coronary ostia or ascending aorta (anterograde cardioplegia).

All patients in the BCS group underwent BCS during the IO and IPO (for 24 hours) periods. BCS included a specialized dual-lumen suction tube that allows a continuous flow of anticoagulants to the suction tip of the catheter, preventing the clotting of the aspirated blood. The vacuum aspiration pressure was kept between 60 mmHg and 100 mmHg to minimize hemolysis during IO and PO aspiration. A solution of 25,000 IU of heparin in 1000 mL of 0.9% saline was added to the blood recovered from the cardiotomy reservoir. The flow of the anticoagulant was adjusted according to the rate of bleeding in the surgical field and the IPO period, and the blood was filtered thereafter. Once a sufficient volume of blood with anticoagulant had reached the cardiotomy reservoir (around 500 mL), processing began by draining the blood from the reservoir into the centrifuge chamber. RBC and saline were then pumped into an infusion bag to be transfused into the patient (reinfusion within up to four hours of blood processing).

After CPB, the heparin was completely neutralized with protamine. To neutralize 1 mg of heparin, 1 mg of protamine sulfate was administered^[16]^. An additional 30% of the total protamine dose was used in the first PO hours to reduce blood loss and the need for blood transfusion^[16]^. The only hemostatic medication used during the procedure was tranexamic acid at a loading dose of 15 mg/kg during anesthetic induction and maintenance at 2 mg/kg/h during surgery for all patients^[17]^.

At the end of the procedure, the patient was extubated in the operation room or recovery room. One hour after extubation, patients were transferred to the intensive care unit, conscious and oriented. In the BCS group, BCS was connected to the mediastinal drain in PO automatic mode.

Blood collection was performed in the preoperative period (24 hours before surgery), IPO period (within the first 24 hours), and on the day of hospital discharge (HD) to evaluate Hb (g/dL) and Ht (%). For all patients, demographic information (sex, age), surgical parameters (type of cardiac surgery, EuroSCORE II, CPB time, aorta cross-clamping time), clinical parameters (preoperative Hb, IPO Hb, HD Hb; preoperative Ht, IPO Ht, HD Ht), clinical outcomes (number of total RBC used in the postoperative period), infection rates (defined as superficial infection with purulent secretion in the sternal surgical wound or lower limb [in cases of myocardial revascularization] with positive culture by swab and deep infection with sternal involvement), PO length of stay, and death within one year were analyzed.

### Statistical Analysis

We performed the Student's *t*-test for continuous variables and the chi-square test for categorical variables. In the absence of the prerequisites of normality and homogeneity, we performed the Mann-Whitney U test. A correlation test was done between age, EuroSCORE, CPB, clinical parameters, and clinical outcomes (total PO RBC and hospital length of stay). A generalized linear model, with Gaussian or gamma distribution (log correction) and identity link function, was used for the clinical outcome of the number of total PO RBC administered and hospitalization stay. A significance level of 5% was adopted, and the RStudio software (version 4.3.2) (packages: dplyer, car, gtsummary, corr, and rstatix) was used.

## RESULTS

Forty-one patients referred to cardiac surgery (BCS = 21, WBCS = 20) were selected according to inclusion/exclusion criteria in three hospitals. The main surgery performed was myocardial revascularization (63%), valve replacement (17%), revascularization + valve replacement (9.8%), aortic surgery (7.3%), and aortic surgery + valve replacement (2.4%). The WBCS group was older than the BCS group (U = 69.5, *P* < 0.001; 64.5 ± 6.5; 56 ± 14). There was no significant difference between both sexes (Table 1). In relation to surgical parameters, there was no significant difference between the groups for EuroSCORE II and CPB time (Table 2). During all the preoperative preparation, patients did not use warfarin, heparin, or other systemic anticoagulant drugs and did not present coagulation disorders or continuous or recurrent systemic sepsis. CPB time was approximately 116.6 ± 4.3 minutes, with an aortic cross-clamping time of 87.4 ± 38.9 minutes and moderate hypothermia (between 28.0℃ and 31.9℃) (Table 2).

**Table 1:** Demographic data between patients in the BCS and WBCS groups.

		Groups		
Variables	Overall N = 41^1^	BCS N = 21^1^	WBCS N = 20^1^	P-value^2^
Age (years)	58.6 ± 10.8	53.1 ± 11.2	64.3 ± 6.8	< 0.001
Sex				
Female	16 (39%)	7 (33%)	9 (45%)	0.444
Male	25 (61%)	14 (67%)	11 (55%)	

1 Mean ± standard deviation; n (%);

2 Wilcoxon rank sum and Fisher's exact tests

BCS=blood cell salvage; WBCS=without the use of blood cell salvage

**Table 2:** Clinical data between patients in the BCS and WBCS groups.

		Groups		
Variables	Overall N = 41^1^	BCS N = 21^1^	WBCS N = 20^1^	P-value^2^
Surgical parameters				
EuroSCORE II	1.9 (2.3)	1.6 (2.3)	2.2 (1.9)	0.575
CPB time (min)	116.6 ± 45.3	124.5 ± 47.9	108.3 ± 42.1	0.266
Aorta cross-clamping time (min)	87.4 ± 38.9	94.9 ± 40.8	79.6 ± 36.2	0.239
Clinical parameters				
Preoperative Hb	13.4 (2.0)	13.9 (2.4)	13.3 (1.6)	0.426
Preoperative Ht	38.9 (7.8)	40.0 (7.8)	36.5 (5.4)	0.540
IPO Hb	11.3 (2.2)	12.0 (1.8)	10.9 (2.5)	0.018
Missing	1	1	0	
IPO Ht	32.1 (7.2)	35.7 (5.6)	30.7 (7.4)	0.009
Missing	1	1	0	
HD Hb	10.3 (1.7)	11.0 (1.5)	10.1 (1.6)	0.047
HD Ht	31.1 (3.9)	32.3 (5.0)	29.9 (3.6)	0.083
Clinical outcome				
Total PO RBC	1.0 (2.0)	0.0 (1.0)	2.0 (0.3)	< 0.001
Length of stay (days)	8.0 (5.0)	7.0 (6.0)	10.0 (5.3)	0.020
Infection	9 (22%)	1 (4.8%)	8 (40%)	0.009
Death	2 (4.9%)	0 (0%)	2 (10%)	0.232

1 Median (interquartile range); n (%);

2 Wilcoxon rank sum and Fisher's exact tests

BCS=blood cell salvage; CPB=cardiopulmonary bypass; EuroSCORE=European System for Cardiac Operative Risk Evaluation; Hb=hemoglobin; HD=hospital discharge; Ht=hematocrit; IPO=immediate postoperative; PO=postoperative; RBC=red blood cell; WBCS=without the use of blood cell salvage

For clinical parameters, there was a significant difference in IPO Hb (U = 288, *P* = 0.018; 12 ± 1.8; 10.9 ± 2.5), IPO Ht (U = 297.5, *P* = 0.009; 35.7 ± 35.6; 30.7 ± 7.4), and HD Hb (U = 286.5, *P* = 0.047; 11 ± 1.5; 10.1 ± 1.6) between the BCS and WBCS groups. The values were higher for patients in the BCS group. No differences were observed in clinical parameters in preoperative Hb, preoperative Ht, and HD Ht values (Table 2).

For clinical outcomes, there was a significantly higher number of total PO RBC administered for patients in the WBCS group compared to the BCS group (U = 76.5, *P* < 0.001; 0.0 ± 1.0; 2.0 ± 0.3) (Table 2). There was a significant decrease in the PO length of stay (U = 121, *P* < 0.020; 7.0 ± 6.0; 10 ± 5.3) and lower infection rates in the BCS group (BCS = 4.8%, WBCS = 40%, *P* = 0.020). After one year of follow-up, two deaths occurred in the WBCS group related to osteomyelitis (Table 2).

### Correlation Analysis

**Fig. 1:**
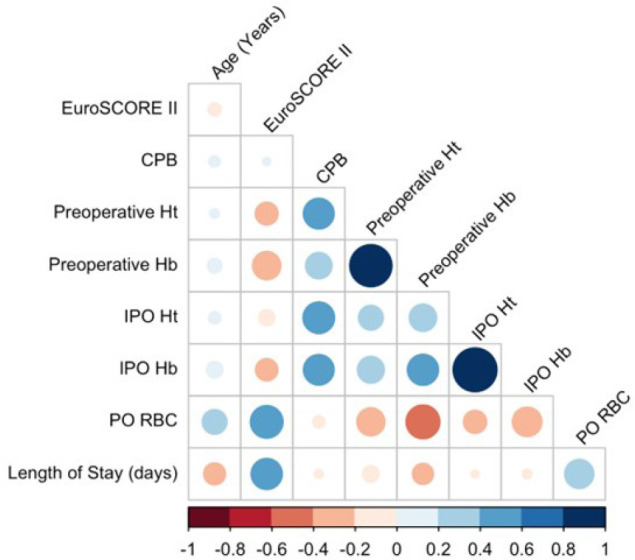
Correlation matrix between age, cardiopulmonary bypass (CBP), European System for Cardiac Operative Risk Evaluation (EuroSCORE) II, clinical parameters, and clinical outcome. Hb=hemoglobin; Ht=hematocrit; IPO=immediate postoperative; PO=postoperative; RBC=red blood cell.

Correlations analyses are shown in Figure 1. The total PO RBC and the length of stay (days) were significantly positively associated (*P* < 0.001). As predicted, the correlation coefficients between the total PO RBC and clinical parameters (preoperative and IPO Hb and Ht) were significantly negatively associated (*P* < 0.001). The negative correlation between preoperative Hb, preoperative Ht, IPO Hb, IPO Ht, and total PO RBC (variable outcome) were -0.49, -0.28, -0.42, and -0.29, respectively. No correlation was observed between PO RBC total, CPB, EuroSCORE, and age.

### Predictors of Total Postoperative Red Blood Cells

For the total PO RBC, we observed that the preoperative Hb (estimate [b] = -0.37; standard error [SE] = 0.12; *P* < 0.006) and IPO Hb (estimate [b] = -0.39; SE = 0.17; *P* < 0.034) influenced the total volume administered. Thus, with the lower preoperative Hb and IPO Hb values, there was an increase of 0.37 and 0.39 in the average number of PO RBC. The IPO Ht values (estimate [b] = 0.13; SE = 0.05; *P* < 0.026) also influenced the number of PO RBC. The higher IPO Ht value increased in 0.13 the PO RBC administered (Table 3).

**Table 3:** Linear regression using the number of total postoperative red blood cells as the outcome, and age, preoperative/IPO Hb, preoperative/IPO Ht, group, and length of stay as predicted.

Characteristic	Beta	95% CI^1^	*P*-value
Age (years)	0.03	-0.01 - 0.06	0.13
Preoperative Ht	0.03	-0.04 - 0.11	0.4
Preoperative Hb	-0.37	-0.62 - -0.11	0.006
IPO Ht	0.13	0.02 - 0.24	0.026
IPO Hb	-0.39	-0.76 - -0.03	0.034
Group			
BCS	-	-	
WBCS	0.92	0.08 - 1.8	0.032
Length of stay	0.05	0.00 - 0.09	0.047

1 Gaussian distribution (AIC: 103.7/R2: 0.61)

BCS=blood cell salvage; CI=confidence interval; Hb=hemoglobin; Ht=hematocrit; IPO=immediate postoperative; WBCS=without the use of blood cell salvage

Being in the WBCS group led to an increase in the average number of PO RBC administered by 0.92 (estimate [b] = 0.92; SE = 0.41; *P* = 0.032), with BCS being the reference group.

The PO length of stay also influenced the PO RBC (estimate [b] = 0.05; SE = 0.02; *P* = 0.047). The longer the length of stay, the higher the increase in the average PO RBC administered, by 0.05. We did not observe an effect of age (*P* = 0.13).

### Predictors of Postoperative Length of Stay

Regarding length of stay, we observed a difference between the BCS and WBCS groups (estimate [b] = 0.73; SE = 0.25; *P* < 0.007), with the BCS group as a reference. Thus, being in the WBCS group led to an increase of 0.73 in the average length of stay (Table 4). Age also influenced length of stay (estimate [b] = -0.03; SE = 0.01; *P* < 0.001). The older patients unexpectedly showed a shorter hospitalization time.

**Table 4:** Linear regression using length of stay as the outcome, and age, preoperative/IPO Hb, preoperative/IPO Ht, groups, and PO RBC as predicted.

Characteristic	Beta	95% CI1	*P* -value
Age (years)	-0.03	-0.05 - -0.01	0.001
Preoperative Hb	-0.11	-0.28 - 0.07	0.2
Preoperative Ht	0.03	-0.02 - 0.08	0.2
IPO Hb	0.21	-0.01 - 0.44	0.063
IPO Ht	-0.02	-0.09 - 0.05	0.5
Group			
BCS	-	-	
WBCS	0.73	0.22 - 1.2	0.007
Total PO RBC	0.18	-0.03 - 0.38	0.088

1Gamma distribution with log (AIC: 63.2/R2: 0.36) BCS=blood cell salvage; CI=confidence interval; Hb=hemoglobin; Ht=hematocrit; IPO=immediate postoperative; PO=postoperative; RBC=red blood cell; WBCS=without the use of blood cell salvage

We did not observe an effect of preoperative Hb, preoperative Ht, IPO Hb, IPO Ht, and total PO RBC in the PO length of stay.

## DISCUSSION

The results indicate that patients in the BCS group had better PO clinical parameters (increase in IPO and HD Hb and IPO and HD Ht) and outcomes (decrease of PO RBC, length of stay, and infection rates) compared to patients in the WBCS group after cardiac surgery with CPB. When analyzing the clinical outcome of PO RBC, the data indicates that low preoperative/IPO Hb, higher length of stay, and being in the WBCS group increased the likelihood of PO RBC administration. In addition, being in the WBCS group increased the length of stay.

Allogeneic blood is a limited therapeutic resource. Current evidence demonstrates blood transfusions excessive use and a decrease in donators due to population aging, resulting in reduced blood supplies worldwide^[6]^. In 2023, the Joint Commission published a study reviewing the prescription of blood components in 15 large American hospitals. It concluded that only 14.52% of prescriptions were adequate, making clear the inadequacy and overuse of blood transfusions even in the best hospital settings^[18]^. Furthermore, blood transfusions are associated to increased morbidity and mortality and higher hospital costs^[2^,^10]^.

During cardiac surgery with CPB, massive blood loss can occur, potentiating the risks of coagulopathies and platelet dysfunction resulting from CPB. In this context, the use of BCS in cardiac surgery can reduce allogeneic blood transfusion by up to 40%^[19]^. In general, the Ht of recovered blood and reinfused from BCS is around 55% to 70%, similar to that found in RBC units from the blood bank^[20]^. It is important to consider that the reinfusion of fresh RBC, from the own patient, without morphological, biochemical, and physiological alterations, as opposed to the ones that occurs from allogeneic blood, significantly favors the supply of oxygen and microcirculation^[19]^. This may also prevent the inflammatory and immunomodulatory effects of excessive allogeneic blood transfusion. Thus, using BCS gives the patient a safer alternative for blood components^[21]^. Our study showed a reduction in the use of PO RBC in the BCS group, maintaining adequate levels of IPO and HD Hb/Ht.

In cardiac surgery, CPB can also induce a systemic inflammatory response that can lead to complications such as acute lung injury and acute kidney injury. At the same time, blood transfusions further exacerbate this inflammatory response and increase morbidity and mortality. A recent study demonstrated that the use of BCS compared to blood transfusions in cardiac surgery was associated with lower interleukin-10 levels, mean blood urea nitrogen, and creatinine levels, as well as 80% reduction in the duration of milrinone use, shorter average time for extubating (in hours), and decreased length of stay by 60%^[22]^.

Although the use of BCS in the IO period has been described, its use in the PO period from the thoracic drain still has limited reports. We identified a recent study that employed BCS in the IPO period (six hours) after cardiac surgery. With the use of BCS in IO and IPO periods, there was a decrease in the incidence of PO atrial fibrillation^[11]^. It is widely recognized that PO atrial fibrillation in cardiac surgery affects between 19% and 50% of patients, with increased length of stay, morbidity, and mortality. Mediastinal bleeding and inflammation are one of the most important factors for atrial fibrillation. Another study in cardiac surgery with the use of BCS from six to 24 hours in the IPO period showed a significant reduction in RBC use without increased infection rates^[15]^.

Our study reinforces the positive results of BCS use, as well as its safety, including a lower infection rate, which is always a concern in the use of PO RBC. The use of BCS in our study did not show an increase in mediastinal hemorrhage, suggesting there is a low concentration of residual heparin in the final recovered blood product.

The World Health Organization released a “Policy Brief” in October 2021, recommending the urgent need to implement the PBM. The goal was to create a sense of urgency for healthcare to implement PBM, through a systematic and multidisciplinary model to improve health and clinical outcomes substantially and increase cost-effectiveness for surgical and non-surgical patients^[10]^. Our study shows that it is possible to advance the practices recommended by the PBM, even in critically ill patients^[22^,^23]^. Cardiac surgery societies have assumed responsibility for bringing these advances to health care with approaches that are increasingly individualized and shared with the patients^[24]^.

### Limitations

Our study was non-randomized. We should also mention the failure to perform serological tests of inflammatory factors to demonstrate the efficacy and safety of BCS use.

## CONCLUSION

In conclusion, our results demonstrated that the BCS use in patients undergoing cardiac surgery with CPB is safe and resulted in better Hb and Ht levels, lower consumption of RBC in the postoperative and HD periods, shorter PO length of stay, and lower infection rates. Furthermore, although there was no statistical difference, the two deaths that occurred after one year of heart surgery were in the group that received a blood transfusion.
